# Long-term follow-up of tuberculosis-destroyed lung patients after surgical treatment

**DOI:** 10.1186/s12890-022-02139-z

**Published:** 2022-09-14

**Authors:** Hongyun Ruan, Fangchao Liu, Yunsong Li, Yuxuan Wang, Dongdong Hou, Xinting Yang, Bin Liu, Teng Ma, Zhidong Liu

**Affiliations:** 1 Department of Cellular and Molecular Biology, Beijing Tuberculosis and Thoracic Tumor Research Institute, Beijing Chest Hospital, Capital Medical University, No 9, Bei guan Street, Tong Zhou District, Beijing, 101149 People’s Republic of China; 2 Beijing Tuberculosis and Thoracic Tumor Research Institute, Beijing Chest Hospital, Science and Technology Office, Capital Medical University, Beijing, People’s Republic of China; 3 Department of Thoracic Surgery, Beijing Tuberculosis and Thoracic Tumor Research Institute, Beijing Chest Hospital, Capital Medical University, No 9, Bei guan Street, Tong Zhou District, Beijing, 101149 People’s Republic of China; 4 Department of Medical Oncology of Peking University Shougang Hospital, Beijing, People’s Republic of China; 5 Department of Tuberculosis, Beijing Tuberculosis and Thoracic Tumor Research Institute, Beijing Chest Hospital, Capital Medical University, Beijing, People’s Republic of China

**Keywords:** Tuberculosis, Damaged lung, Surgical treatment, Modified British medical research council (mMRC), Living status

## Abstract

**Background:**

To monitor dypsnea and mortality at 5 and 10 years, respectively, after surgical treatment of tuberculosis-destroyed lung (TDL) patients.

**Methods:**

TDL patients treated surgically at Beijing Chest Hospital from November 2007 to June 2019 were monitored in this observational study. Follow-up assessments of respiratory function indicators and survival conducted 5 and 10 years post-surgery led to patient grouping based on mMRC score into a dyspnea group (mMRC ≥ 1) and a non-dyspnea group (mMRC = 0). Cox regression analysis detected effects of patient demographics, clinical characteristics, surgical factors and respiratory function on 5 year post-surgical survival.

**Results:**

By study completion (June 30, 2020), 32 of 104 patients were lost and 72 completed follow-up for a study total of 258.9 person-years. 45 patients (62.5%, 45/72) had mMRC scores of 0, while 12 (16.7%, 12/72), 21 (36.2%, 21/58) and 27 (60.0%, 27/45) patients exhibited dyspnea by 1, 3 and 5 years post-surgery, respectively. Low lung carbon monoxide diffusion score (DLCO% pred) and scoliosis contributed to dyspnea occurrence.

**Conclusions:**

Most TDL patients lacked subjective dyspnea signs post-surgery, while dyspnea rates increased with time. Preoperative low lung diffusion function and Scoliosis were associated with factors for postoperative dyspnea. Surgical treatment increased TDL patient survival overall.

## Introduction

Tuberculous lung destruction (TDL) is characterized by diffuse structural damage to the lungs and a basic loss of respiratory function, which is very common in populations in developing countries [1, 2]. Hemoptysis, low fever, yellow sputum, chest tightness, and shortness of breath are the common symptoms in the patients. When developed the life-threatening hemoptysis symptom, surgical treatment is required [2–4]. Some patients with TDL have multidrug-resistant tuberculosis (MDR-TB) or extensively drug-resistant tuberculosis (XDR-TB), which could be treated surgically [4–6]. By the removal of ineffectively ventilated lung tissue, the ventilation/perfusion ratio could be improved, which relieves the patient’s dyspnea [7, 8]. Studies have shown that surgical treatment of TDL is effective, with complication rates is 9.6–45.7% [8–10]. Most previous TDL studies have focused on postoperative complications and mortality [11–15]. However, few studies focused on long-term TDL patient post-surgical prognosis based on long-term survival rates or clinical cure rates [16, 17]. In this study, we used Modified British Medical Research Council (mMRC) scores to evaluate TDL patient dyspnea and mortality for at least 12 months. The objective was to describe long-term outcomes of pulmonary resection surgical treatment of TDL patients by focusing on dyspnea incidence, survival of patients with severe dyspnea and factors contributing to long-term patient outcomes.

## Materials and methods

### Study subjects

Patients with TDL who underwent surgical treatment in the Department of Thoracic Surgery, Beijing Chest Hospital, Capital Medical University from November 2007 to June 2019 received follow-up for at least one year through June 30, 2020. The total follow-up time was 258.9 person-years and the median follow-up time was 3.85 years. The patient cohort included 44 males and 28 females of ages within the range of 37.9 ± 12.5 years. Postoperative dyspnea was defined as dyspnea with an mMRC score of ≥ 1 that first occurred in TDL patients between the time of postoperative discharge to the time of follow-up completion. Inclusion criteria included: (i) preoperative arterial blood gas analysis without respiratory failure; (ii) postoperative pathomorphology consistent with tuberculosis; (iii) surgical treatment performed according to the surgical indication of TDL. Exclusion criteria included: (i) bronchial asthma; (ii) chronic obstructive pulmonary disease; (iii) Interstitial lung disease; (iv) complicated disease due to malignant tumor; (v) chronic cardiac and rena insufficiency; (vi) chronic diseases identified during follow-up that directly affected mMRC scores.

The study design complied with the Helsinki Research Ethics Statement and was approved by the Ethics Committee of Beijing Chest Hospital (2018) Clinical Review No (43).

### Data and methods

Demographic and general clinical data included age at onset, sex, body mass index (BMI) category (BMI < 18.5 underweight, BMI 18.5–24.9 normal, BMI ≥ 25 obese), smoking status, major complications (hypertension, coronary heart disease, diabetes), location of TDL disease (left, right) and presence or absence of chronic pulmonary aspergillosis and/or non-tuberculous mycobacterial infection.

The presence versus absence of lesion(s) in the contralateral lung (non-TDL lung) and of spinal scoliosis were determined using chest CT scans. The degree of scoliosis was assessed by measuring contralateral curvature angles (Cobb angles), with Cobb angles of ≤ 5 scored as normal and scores of > 5 scored as curved. A Jaeger Master Screen PFT device was used to measure lung function. Diffusing capacity of the lungs for carbon monoxide was measured using the single-breath method. Lung function data included forced vital capacity of predicted value (FVC% pred) and lung diffusion capacity for carbon monoxide of predicted value (DLCO% pred).

Surgical information collected for each patient reflected the extent of removal of lung lesions based on type of surgical intervention that included left or right lobectomy, lobectomy with pleural clearance and the left or right pneumonectomy with pleural clearance. Thoracoscopic surgery was performed in one case, while thoracotomy was performed in all other cases.

### Follow-up

Face-to-face and telephone follow-up surveys were administered to TLD patients by trained health care practitioners from November 2008 to June 2020, for a median follow-up duration of 4.8 years. During the post-operative follow-up period, monitoring was conducted of both readmitted patients and those who were never readmitted in order to collect patient information with regard to postoperative antituberculous chemotherapy, mMRC score and survival status. Definitions of mMRC scores were as follows: 0, I have difficulty breathing only during strenuous exercise; 1, I get shortness of breath from walking briskly or climbing hills on flat ground; 2, Because of my shortness of breath, I walk more slowly on flat ground than my peers or need to stop to rest; 3, I need to stop and catch my breath after about 100 m or a few minutes on flat ground; 4, I had severe breathing difficulties that prevented me from leaving the house or from putting on or taking off my clothes. Based on mMRC scores, patients were divided into two groups: a non-dyspnea group (mMRC = 0) and a dyspnea group (mMRC ≥ 1). In this study, censored data were defined as an absence of dyspnea (n = 45) or loss of the patient to follow-up (n = 0) by June 2020. Re-hospitalization status and TLD surgery-associated factors responsible for readmittance included secondary bronchopleural fistula, repeated hemoptysis, infection, empyema, etc. as confirmed by two senior chief physicians. Regarding post-surgical use of anti-tuberculosis chemotherapy drugs, most patients complied with doctors’ recommendations and took their anti-tuberculosis chemotherapy drugs on time and completed the chemotherapy treatment course by 6–12 months post-surgery. However, a few patients did not take anti-tuberculosis drugs for economic or personal reasons.

### Quality control

The follow-up plan and questionnaire were reviewed and verified by respiratory and thoracic surgery experts, while health doctors supervised the follow-up program. Face-to-face or telephone follow-up interviews to collect patient data were conducted by trained health care doctors who followed a standard procedure outlined in the study manual and used questionnaires that had been reviewed by the quality control team.

### Statistical analysis

Categorical variables were expressed as numbers of cases and case proportions. The median and interquartilerange (IQR) were used to depict mMRC scores. The Wilcoxon-Sign rank sum test was used to compare differences of mMRC scores before and after surgical treatment of TDL patients, with compositions of mMRC groups before and after surgery compared using the McNemar Test. Results obtained using Cox models were interpreted based on dyspnea outcome as defined as the first occurrence of mMRC ≥ 1 detected during follow-up to June 2020; censored data was defined as no occurrence of dyspnea (mMRC ≥ 1, n = 45) or loss of patient to follow-up (n = 0) by June 2020. We incorporated risk factors (old age/low preoperative FEV1) based on risks reported previously [8, 13] and incorporated statistically significant variables revealed by the univariate Cox model into the multivariate Cox regression model. The stepwise variables selection method was used to screen for factors associated with dyspnea and Kaplan–meier method was used to plot the survival curve of patients with dyspnea according to mMRC ≥ 1 score. statistical analyses were performed using SPSS21.0 (SPSS, INC., Chicago, IL, USA).

## Results

**Fig. 1:**
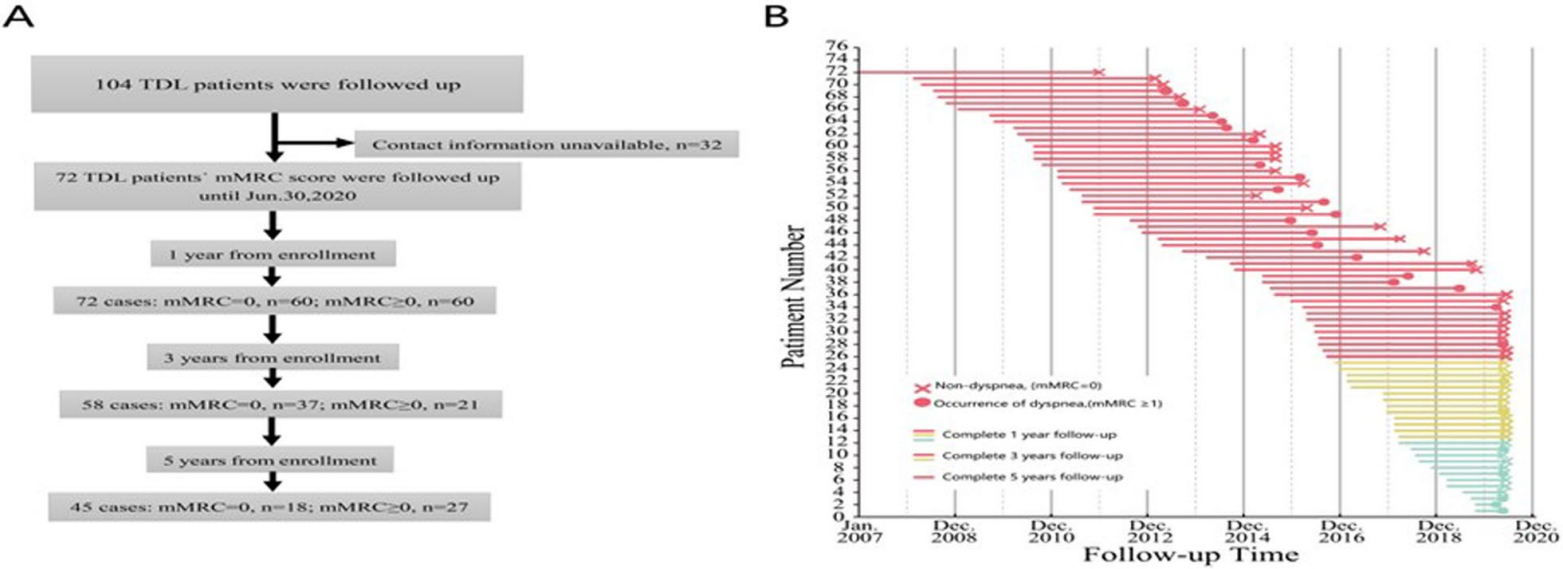
**A** Follow-up Flow Chart showing the duration of follow-up for each participant. **B** Follow-up of 72 patients was frequent

Follow-up monitoring of a total of 104 TDL patients who had undergone pulmonary resection surgery was conducted until June 30, 2020. Of the 104 cases, 32 cases were excluded from analysis, for a loss rate of 30.8% (32/104). For the 72 patients who completed follow-up, total follow-up times ranged from 1 to 5 years and the median follow-up time was 3.85 years (Fig. 1A).

Among 72 patients, 62.5% (45/72) of patients had no dyspnea (mMRC scores of 0) and received follow-up monitoring for 158.3 person-years. 37.5% (27/72) patients had varying degrees of dypsnea (with mMRC scores ≥ 1) and received follow-up monitoring for 100.6 person-years (Table 1). Proportions of patients with mMRC scores ≥ 1 in the first, third and fifth years after surgery were 12 (16.7%, 12/72), 21 (36.2%, 21/58) and 27 (60.0%, 27/45), respectively, of whom 2 patients received long-term home oxygen therapy (LTOT) for dyspnea due to secondary bronchopleural fistula (Fig. 1B).

**Table 1:** Follow up of 72 patients with TDL on postoperative mMRC score

mMRC score	Follow up time (person-year)	Year post-operative (n = 72, %)	Year post-operative (n = 58, %)	Year post-operative (n = 45, %)
0	158.3	60 (83.3)	37 (63.8)	18 (40.0)
≥ 1	100.6	12 (16.7)	21 (36.2)	27 (60.0)

*TDL* Tuberculosis destroyed lung; *mMRC* Modified British medical research council

Preoperative mMRC scores of 72 TDL patients were higher than their respective postoperative mMRC scores (*P* = 0.006). The difference in preoperative and postoperative numbers of patients in mMRC groups (mMRC = 0 vs. mMRC ≥ 1) was not statistically significant (*P* = 0.25). However, the preoperative proportion of patients within the mMRC ≥ 3 group (12.5%) was much greater than the corresponding postoperative proportion of patients in this group (0%) (Table 2).

**Table 2:** Comparison of mMRC scores before and after operation in patients with TDL

mMRC	Preoperative (n = 72)	Postoperative (the day of discharge) (n = 72)	*P* value	5 year post-operative (n = 45)
mMRC (median, IQR)^a^	1 (0–1)	0 (0–1)	0.006	0 (0–1)
*mMRC group, n (%)*^b^				
mMRC = 0	21 (29.2)	24 (33.3)	0.250	18 (40.0)
mMRC = 1	37 (51.4)	38 (52.8)		14 (31.1)
mMRC = 2	5 (6.9)	10 (13.9)		10 (22.2)
mMRC = 3	9 (12.5)	0 (0)		2 (4.5)
mMRC = 4	0 (0)	0 (0)		1 (2.2)

*TDL* Tuberculosis destroyed lung; *mMRC* Modified British medical research council; *IQR* Inter quartile range

a: Wilcoxon Signed rank-sum test; b: McNemar Test (mMRC = 0 vs. mMRC ≥ 1)

**Fig. 2:**
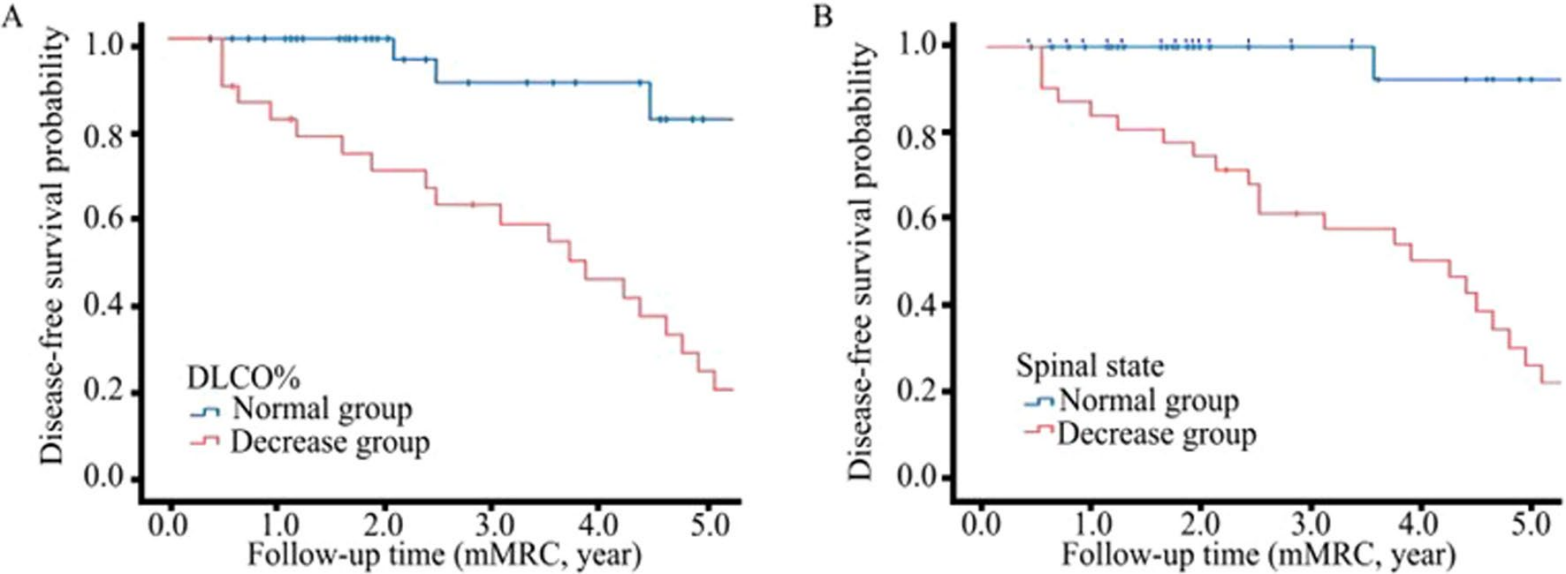
Kaplan–Meier curve showing effect of postoperative dyspnea on survival of 72 patients with tuberculous lung damage

The results of univariate Cox regression showed that the incidence rate of cases with both TDL and scoliosis was higher than that of TDL cases without scoliosis (*P* = 0.001). In addition, the number of cases in the patient group with DLCO% pred < 80% was higher than the corresponding number in the patient group with DLCO% pred ≥ 80% (*P* = 0.044) (Table 3). Multivariate Cox regression analysis revealed that a value of DLCO% pred < 80% (HR: 2.735, 1.135–6.588) and scoliosis (HR: 4.467, 1.900–10.500) were risk factors for postoperative dyspnea (Table 4). The statistically significant difference between Kaplan–Meier curves stratified by DLCO% pred and scoliosis was also showed in Fig. 2.

**Table 3:** Univariate Cox analysis of dyspnea in patients with tuberculous lung damage

Variables	Follow-up time Person-years	Incidence rate, per 10 person-years	Total (n, %)	mMRC = 0 (n = 45, %)	mMRC ≥ 1 (n = 27, %)	HR (95%CI)	*P* value
Sex							
Male	92.5	1.1	28 (38.9)	18 (64.3)	10 (35.7)	Ref	
Female	166.4	1.0	44 (61.1)	27 (61.4)	17 (38.6)	0.787 (0.358–1.729)	0.551
Age group (years)							
< 60	242.4	1.0	67 (93.1)	43 (64.2)	24 (35.8)	Ref	
≥ 60	16.5	1.8	5 (6.9)	2 (40.0)	3 (60.0)	0.826 (0.240–2.845)	0.762
BMI (kg/m^2^)							
< 18.5)	38.4	1.3	12 (16.7)	7 (58.3)	5 (41.7)	Ref	
18.5–24.9	191.0	1.0	52 (72.2)	33 (63.5)	19 (36.5)	0.630 (0.234–1.694)	0.360
≥ 25	29.5	1.0	8 (11.1)	5 (62.5)	3 (37.5)	0.397 (0.093–1.693)	0.212
Smoking history							
No	230.2	1.1	62 (86.1)	37 (59.7)	25 (40.3)	Ref	
Yes	28.7	0.7	10 (13.9)	8 (80.0)	2 (20.0)	0.712 (0.168–3.016)	0.645
Comorbidities^a^							
No	209.8	1.1	57 (79.2)	34 (59.6)	23 (40.4)	Ref	
Yes	49.1	0.8	15 (20.8)	11 (73.3)	4 (26.7)	1.313 (0.443–3.890)	0.623
mMRC							
0	80.1	1.2	20 (27.8)	10 (50.0)	10 (50.0)	Ref	
≥ 1	178.8	1.0	52 (72.2)	35 (67.3)	17 (32.7)	0.486 (0.170–1.389)	0.178
Site of TDL							
Left lung	203.9	0.9	56 (77.8)	37 (63.2)	19 (32.8)	Ref	
Right lung	55.0	1.5	16 (22.2)	8 (50.0)	8 (50.0)	1.874 (0.817–0.296)	0.138
Combined with other pathogenic bacteria							
No	183.5	1.0	50 (69.4)	31 (62.0)	19 (38.0)	Ref	
CPA or NTM	75.4	1.1	22 (30.6)	14 (63.6)	8 (36.4)	1.263 (0.550–2.899)	0.583
Chest CT							
Number of contralateral lesions							
< 2	158.2	1.1	42 (58.3)	25 (59.5)	17 (40.5)	Ref	
≥ 2	100.7	1.0	30 (41.7)	20 (66.7)	10 (33.3)	1.132 (0.506–2.534)	0.762
Spinal							
Cobb angles ≤ 5°	156.7	0.6	40 (55.6)	30 (75)	10 (25)	Ref	
Cobb angles > 5°	102.2	1.7	32 (44.4)	15 (46.9)	17 (53.1)	4.628 (1.953–10.964)	< 0.01
Lung function							
FVC, L	–	–	–	2.4 ± 0.7	2.2 ± 0.6	0.621 (0.245–1.570)	0.314
FVC%							
≥ 80% pred	39.6	0.5	11 (15.3)	9 (81.8)	2 (18.2)	Ref	
< 80% pred	219.3	1.1	61 (84.7)	36 (59.0)	25 (41.0)	2.183 (0.501–9.506)	0.298
FEV_1_, L	–	–	–	1.9 ± 0.6	1.7 ± 0.5	0.457 (0.157–1.332)	0.151
FEV_1_/FVC, %	–	–	–	78.1 ± 11.6	74.1 ± 11.6	0.970 (0.922–1.020)	0.233
DLCO, L	–	–	–	6.0 (4.8,6.9)	5.1 (4.3,7.2)	0.869 (0.646–1.168)	0.352
DLCO%							
≥ 80%pred	163.3	0.4	44 (61.1)	37 (84.1)	7 (15.9)	Ref	
< 80%pred	95.6	2.1	28 (38.9)	8 (28.6)	20 (71.4)	2.794(1.168–6.683)	0.021
Albumin, g/L	–	–	–	39.4 ± 7.7	37.5 ± 5.2	0.957 (0.887–1.033)	0.257
Albumin							
Normal	203.7	1.0	54 (75.0)	34 (63.0)	20 (37.0)	Ref	
Decreased	55.2	1.3	18 (25.0)	11 (61.1)	7 (38.9)	1.557 (0.654–3.706)	0.317
Hemoglobin, g/L	–	–	–	119.4 ± 20.7	122.3 ± 18.4	1.008 (0.983–1.033)	0.541
Hemoglobin							
Normal	240.5	1.1	67 (93.1)	41 (61.2)	26 (38.8)	Ref	
Anemia	18.4	0.5	5 (6.9)	4 (80.0)	1 (20.0)	0.733 (0.097–5.560)	0.764
Blood glucose^b^							
PaO_2,_ Torr	–	–	–	98.3 (88.6,111)	95.0 (86.7,111.8)	0.994 (0.979–1.009)	0.405
PaO_2_							
Normal	175.7	0.9	50 (92.6)	35 (70.0)	15 (30.0)	Ref	
Decreased	16.9	1.2	4 (7.4)	2 (50.0)	2 (50.0)	1.607 (0.357–7.236)	0.537
PaCO_2,_ Torr	–	–	–	42.0 (40.0,45.3)	43.4 (40.6,46.3)	1.088 (0.926–1.277)	0.305
PaCO_2_							
Normal	137.1	0.8	39 (72.2)	28 (71.8)	11 (28.2)	Ref	
Decreased	55.5	1.1	15 (27.8)	9 (60.0)	6 (40.0)	1.157 (0.424–3.153)	0.776
Surgical excision range							
Lobectomy of lungs	89.9	1.1	25 (34.7)	15 (60.0)	10 (40.0)	Ref	
Pneumonectomy with or without pleura	169.0	1.0	47 (65.3)	30 (63.8)	17 (36.2)	0.657 (0.297–1.454)	0.300

*BMI* Body mass index; *CPA* Chronic pulmonary aspergillosis; *FVC%* Forced vital capacity of predicted; *DLCO%* Lung diffusion capacity of predicted; *HR* Hazard ratio

a: Comorbidities including hypertension diabetes and coronary heart disease; b: Preoperative arterial blood gas analysis was performed in 54 patients

**Table 4:** Multivariate Cox analysis of dyspnea after tuberculosis lung damage

Variables	Total	mMRC ≥ 1	*P* value	aHR	95%CI
*DLCO%*					
≥ 80% pred	44	7	–	Ref	–
< 80%pred	28	20	0.025	2.735	1.135–6.588
*Spinal scoliosis*					
Cobb angles ≤ 5	40	10	–	Ref	–
Cobb angles > 5°	32	17	0.001	4.467	1.900–10.500

*mMRC* Modified British medical research council; *DLCO%* Lung diffusion capacity; *aHR* Adjusted hazard ratio calculated by multivariate Cox regression; spine normal, Cobb angles ≤ 5; scoliosis, Cobb angles > 5°

During the follow-up period after TDL surgery, 5.5% (4/72) of patients were hospitalized for a second time, including 2 patients with bronchopleural fistula, 1 patient with relapse due to Mycobacterium tuberculosis infection and 1 patient with recurrent hemoptysis. Meanwhile, 94.4% (68/72) of patients were treated with antituberculotic drugs for treatment durations ranging from 0.1 to 72 months and a median treatment duration of 8 months (Table 4).

By the end of the follow-up period, 9 patients had died, with 3 patients dying on the 5th, 21st and 65th day after surgery due to multiple organ failure caused by excessive blood loss after thoracotomy for hemostasis. Another 3 patients died at home at 1, 3 and 5 years after surgery due to unknown causes, 2 patients died of lung cancer by 3 and 5 years after surgery and 1 patient died of pneumonia 9 years after surgery. Among the 9 patients who died, 5 patients were afflicted with both TDL and pulmonary Aspergillus infection, 1 with rheumatoid arthritis, and 1 with compulsory spondylitis (Table 5).

**Table 5:** Survival of patients with tuberculous lung damage during follow-up

Number of follow-up cases (n = 72)	Follow up time (person-year)	Follow-up of 1 year (%)	Follow-up of 3 years (%)	Follow-up of 5 years (%)	Follow-up of 10 years (%)	Rehospitalization (%)	Postoperative anti-tuberculous chemotherapy drugs (%)
Survival	258.9	72 (98.6)	58 (95.1)	45 (90.0)	12 (66.7)	4 (5.6)	68 (94.4)
Death	31	1 (1.4)	3 (4.9)	5 (10.0)	6 (33.3)	–	–

Binary logistic regression analysis revealed that male sex (OR: 12.6, 1.491–106.016), age > 60 years old (OR: 10.7, 2.168–53.002), preoperative acute massive hemoptysis (OR: 8.5, 1.531–47.182) and postoperative respiratory infections (OR: 3.927, 1.301–11.856) were statistically significant risk factors for postoperative death in TDL patients (Table 6).

**Table 6:** Binary logistic analysis of risk factors for death after tuberculosis lung damage

Parameter	Male	> 60(years)	Smoking history	BMI < 18.5 kg/m^2^	Combined with chronic aspergillus infection	Comorbidities	Acute massive hemoptysis operation	Postoperative respiratory failure
Wald	5.415	8.462	0.052	0.124	0.029	0.705	5.989	5.887
*P* value	0.02	0.004	0.819	0.725	0.865	0.401	0.014	0.015
aOR	12.571	10.72	0.775	0.743	1.136	1.9	8.5	3.927
95%CI	1.491–106.016	2.168–53.002	0.087–6.880	0.142–3.883	0.260–4.961	0.425–8.499	1.531–47.182	1.301–11.856

*BMI* Body mass index; *OR* Odds ratio; *CI* Confidence interval

## Discussion

TDL was a secondary diagnosis in 1.3% of patients with tuberculosis [18]. Damaged lungs are prone to serious complications, including haemorrhage, empyema, secondary fungal infections and sepsis that can be treated by installation of a pulmonary systemic-shunt [19]. Severe hemoptysis can be treated effectively with surgery [2–4], which has also been shown to increase TDL patient long-term survival rates [11–15]. Results of one study demonstrated survival rates of 95% and 88% at 1 and 5 years after TDL surgery, respectively [17], while another study reported 5 year and 10 year survival rates of 94% and 87%, respectively [16]. Our results showed that survival rates at 1, 3, 5 and 10 years after TDL surgery were 98.6%, 95.1%, 90.0% and 66.7%, respectively, with improvement of dyspnea observed after surgery. Thus, these results suggest that TDL surgery can improve long-term survival rates, while also alleviating dyspnea.

In this study, TDL patients who received surgical treatment exhibited reduced rates of dyspnea (as compared to the preoperative rate) prior to discharge and at the end of long-term follow-up. Analysis of the possible mechanism underlying beneficial effects associated with surgical removal of diseased nonfunctional lung tissue revealed that nonfunctional lung tissue may have interfered with effective ventilation such that removal of these tissues led to intra-pulmonary arteriovenous shunting of blood that improved oxygenation and alleviated dyspnea. Meanwhile, persistence or emergence of postoperative dyspnea in some patients may have been associated with numerous diverse factors that caused further reduction of healthy lung tissue volume and degenerative changes that decreased lung function, warranting further investigation.

Our results suggest that scoliosis and preoperative DLCO% pred values < 80% are independent risk factors for postoperative dyspnea. The role played by scoliosis in perpetuating dyspnea after surgery may be due to the fact that TDL pneumonectomy cannot completely reverse scoliosis-induced impaired pulmonary ventilation and severely impaired restrictive ventilation. Meanwhile, low preoperative DLCO% values indicated the presence of TDL-induced severe and/or extremely severe pulmonary ventilation/perfusion dysregulation that could not be completely alleviated by surgical resection of lesions. Thus, in these patients surgery could not completely correct dysregulation of the pulmonary ventilation/perfusion ratio to adequately restore alveolar gas exchange, leading to persistence of postoperative dyspnea of variable severity. Nevertheless, very few patients with scoliosis required a long course of home oxygen therapy.

Our findings suggest that benefits and risks of surgery should be carefully assessed in patients with TDL with scoliosis and preoperative DLCO% pred values of < 80%. Moreover, enhanced pulmonary rehabilitation should be provided to this group of TDL patients after surgery.

In our follow-up observation cohort, a larger proportion of postoperative deaths occurred in males than females, accounting for 88.9% (8/9) and 55.6% (5/9) of pulmonary TB cases with coinfections with Aspergillus or non-mycobacteria, respectively. This result may reflect the fact that male TB patients are more likely to contract Aspergillus and non-mycobacterial infections than are females, due to greater exposures of males to the surrounding environment [20]. In Asia and Africa, 15.4% of TB cases are coinfected with Aspergillus or non-tuberculous mycobacteria, with Aspergillus coinfection significantly increasing risk of death [1]_._ In addition, our recent study demonstrated that TDL accompanied by chronic pulmonary aspergillosis was associated with an increase in number of serious postoperative complications in men [15]. Therefore, short- and long-term risks and benefits of surgery for men, especially those with pulmonary aspergillosis and/or non-pulmonary mycobacterial infection, need to be carefully evaluated.

This study had several limitations. First, it was a single-center observational follow-up study that utilized a patient cohort comprised of a limited number of cases, which may have led to biased results. Second, cases were monitored throughout a long-term follow-up period to collect data related to survival, respiratory function, rehospitalization and postoperative anti-TB treatment status. However, postoperative complications, postoperative psychological effects, such as anxiety and depression, as well as nutrition, exercise and other lifestyle factors and effects of confounding factors on respiratory function were not included in the analysis, which may have led to biased study results.

## Conclusions

Most patients without signs of subjective dyspnea after TDL surgery enjoy good quality of life. However, as post-operative survival time increases, the number of patients with dyspnea increases, although very few patients require home oxygen therapy. Notably, preoperative low pulmonary diffusion function and scoliosis were associated with factors for postoperative dyspnea. Thus, for TDL patients with preoperative scoliosis, DLCO% pred < 80%, and male sex, benefits and risks of TDL surgery should be fully evaluated preoperatively, while enhanced pulmonary rehabilitation training should be provided postoperatively to this group of patients.

## Data Availability

The datasets used and/or analysed during the current study available from the corresponding author on reasonable request.

## Abbreviations

TDL: Tuberculosis-destroyed lung
mMRC: Modified British medical research council
MDR-TB: Multidrug-resistant tuberculosis
XDR-TB: Extensively drug-resistant tuberculosis
BMI: Body mass index
FVC% pred: Forced vital capacity of predicted
DLCO% pred: Lung diffusion capacity for carbon monoxide of predicted
CRP: C-reaction protein
OR: Odds ratio
CI: Confidence interval
